# Consultations on driving in people with cognitive impairment in primary care: A scoping review of the evidence

**DOI:** 10.1371/journal.pone.0205580

**Published:** 2018-10-15

**Authors:** Carol Sinnott, Tony Foley, Justin Forsyth, Kathleen McLoughlin, Linda Horgan, Colin P. Bradley

**Affiliations:** 1 THIS Institute (The Healthcare Improvement Studies Institute), University of Cambridge, Cambridge, United Kingdom; 2 Department of General Practice, University College Cork, Cork, Ireland; 3 Department of Occupational Science and Occupational Therapy, University College Cork, Cork, Ireland; University of Malaya, MALAYSIA

## Abstract

**Objectives:**

To review the empirical evidence on approaches used by Primary Care Physicians (PCPs) in fitness to drive (FtD) consultations with people living with cognitive impairment.

**Design:**

Scoping review of empirical literature focused on primary studies of any design.

**Setting:**

Primary care practice.

**Participants:**

PCPs or their equivalent and/ or individuals with cognitive impairment across the spectrum of mild cognitive impairment to dementia.

**Measurements:**

Systematic search of Medline, Cinahl, PsychINFO, Academic Search Complete, Psychological and Behavioural Sciences Collection, SocIndex and Social Sciences FT were conducted. Records screened by two reviewers against agreed inclusion criteria. Mixed studies (qualitative and quantitative) were synthesized within overarching themes.

**Results:**

Eighteen studies met our inclusion criteria. Synthesized data showed PCPs have mixed feelings on the appropriateness of their role in FtD assessments, with many feeling particularly uncomfortable and lacking confidence in the context of possible cognitive impairment. Reasons include lack of familiarity with legal requirements and local resources; fear of damaging the doctor-patient relationship; and impact on the patient’s quality of life. Patients voiced their desire to maintain agency in planning their driving cessation. Studies evaluating pragmatic educational programmes suggest these can improve physician confidence in FtD consultations.

**Conclusion:**

The increasing number of older people affected by cognitive impairment, for whom driving may be a concern, has implications for primary care practice. Addressing the reasons for PCPs lack of comfort in dealing with this issue is essential in order for them to better engage in, collaborative discussion with patients on plans and preferences for driving cessation.

## Introduction

Evidence suggests that, on average, most older people will outlive their driving expectancy by 7–10 years [1] yet it remains rare for a person to plan ahead for the day when they will cease driving [2]. Driving is a complex task requiring a high level of cognitive functioning [3]. As we age, a broad spectrum of cognitive ability emerges ranging from normal cognitive functioning at one end to dementia at the other. While a diagnosis of dementia does not mean that a person must immediately stop driving, as the disease progresses the ability to drive safely is eventually lost and driving cessation decisions must be made [3].

With the increased detection and diagnosis of dementia, addressing fitness to drive (FtD) and helping patients with cognitive impairment plan for driving cessation is becoming an increasingly frequent aspect of primary care practice [3, 4]. However, the transition to driving retirement can be difficult for patients, and primary care physicians (PCPs) perceive it as a problematic topic that can upset the doctor-patient relationship, especially in the context of cognitive impairment [5, 6]. Unfortunately, cognitive impairment itself is a topic PCPs are also sometimes reluctant to broach with their patients; this reluctance stems from uncertainty about differentiating significant cognitive impairment from natural ageing, and trying to avoid causing patient anxiety about a condition associated with a bleak outlook [7]. However, concerns about cognition cannot be ignored in the context of a FtD consultation. Ideally cognitive impairment would be discussed between patient and PCP at an early stage in the condition, allowing thorough assessment and planning for the future if required. However, until the rates of detection, diagnosis and disclosure of cognitive impairment improve, there is a need to equip PCPs with nuanced communication techniques to deal with both of these sensitive topics in the one consultation [8].

A first step is to acquire an understanding of where and why problems arise in these consultations; what patients’ desire from their PCPs in these consultations; and what has worked to support PCPs in these consultations in the past. While studies from a range of countries have elicited PCP and patient views on FtD assessment [9–11], a synthesis of these studies has the potential to achieve a broader understanding of the challenges than a single empirical study. Therefore, the aim of this study is to describe and synthesize existing empirical evidence on both primary care physicians’ and patients’ experiences of FtD consultations in the setting of cognitive impairment in primary care.

## Method

We chose the scoping review framework proposed by Arksey and O’Malley [12] rather than a conventional systematic review approach as we anticipated that studies relevant to our interest would potentially use mixed study designs (qualitative and quantitative), and we sought to map out the existing evidence identify gaps in the evidence base where further research may be advantageous. Scoping reviews follow five stages. In stage one, we identified our research question as having three components: i) empirical research on ii) primary care consultations with patients about iii) the issue of FtD in the context of cognitive impairment (across the spectrum from mild cognitive impairment to dementia). Stage two was identification of relevant studies. We developed our search strategy by drawing on the search terms used in recent systematic reviews on dementia/cognitive impairment [13, 14], General Practice [15, 16] and driving [17, 18]. An example of the Medline search strategy is available in S1 Table. We searched seven databases (Medline, Cinahl, PsychINFO, Academic Search Complete, Psychological and Behavioural Sciences Collection, SocIndex and Social Sciences FT) for English language papers from inception until 1^st^ December 2016. The specific search dates for each database are provided in the S2 Table. Grey literature was sought on Google, Google Scholar and websites of international dementia organisations for professionals and patients.

Stage 3 is study selection. We imported all citations from our search into an online platform (Covidence) for systematic literature reviews. Titles were screened by one reviewer (KMcL), removing those obviously not relevant. Two reviewers (KMcL and CS) independently screened the remainder by title and abstract, and selected all potentially relevant citations for full text review. Full text papers were reviewed independently by two reviewers (KMcL and CS) against our inclusion criteria (Table 1). While inclusion and exclusion criteria were set *a priori*, we articulated the application of these criteria at this stage through a series of team meetings. For example, we identified many studies examining dementia care or FtD assessment more broadly but decided to include only those papers with findings that, at least in part, addressed both communication between patient and PCP on FtD in the setting of cognitive impairment. We included studies on patient views, as we felt these studies could usefully inform PCPs’ communication techniques in the consultation. Discrepancies were discussed with a third reviewer (CB). Reference and citation lists of included papers were manually searched for other relevant papers.

**Table 1:** Inclusion and exclusion criteria.

Included Studies	Excluded Studies
Primary research (i.e. has generated empirical evidence). Focused on the medical assessment of fitness to drive for people with cognitive impairment, across the spectrum from mild cognitive impairment to dementia. Focused on consultations and communication between people with cognitive impairment and/or their main caregiver with Primary Care Physicians or their equivalent.	Not primary studies (e.g. book reviews, editorials, opinion pieces, expert advice) or not reporting primary empirical findings. Focused on fitness to drive amongst populations with transient cognitive impairment or other medical conditions. Focused on development or validation of psychometric assessment of fitness to drive, or assessment in settings other than general practice, without data on consultation and communication between patients and their GPs or equivalent. Focused on fitness to drive assessment without reference to cognitive impairment, or on cognitive assessment without any reference to driving.

In Stage 4, we extracted data from each included study. For papers that addressed dementia care or FtD assessment more broadly, we extracted only that data which related specifically to FtD in the setting of cognitive impairment. To achieve our aim of mapping all evidence relevant to this literature, we sorted relevant data (both qualitative and quantitative) into a broad inductive analytical framework, and then coded this material into conceptual themes in multiple iterative moves. As is the norm in scoping reviews [12], we did not undertake a comprehensive appraisal of the methodological quality of included studies but we did ensure studies were not “fatally flawed” using the Mixed Methods Appraisal Tool [19, 20]. This tool has been designed for the appraisal stage of complex systematic literature reviews that include qualitative, quantitative and mixed methods studies (mixed studies reviews).

In stage 5, we collated and summarized our results. To present an overview of all material reviewed, we combined our qualitative and quantitative data in a single narrative synthesis, aligning quantitative data with themes evident in the qualitative studies [12, 19]. For quantitative studies, we present findings using proportions and percentages and for qualitative studies present illustrative quotes (in italics). For pre/post studies, we present baseline findings first and then describe the impact of the interventions in a separate section.

## Results

**Fig 1:**
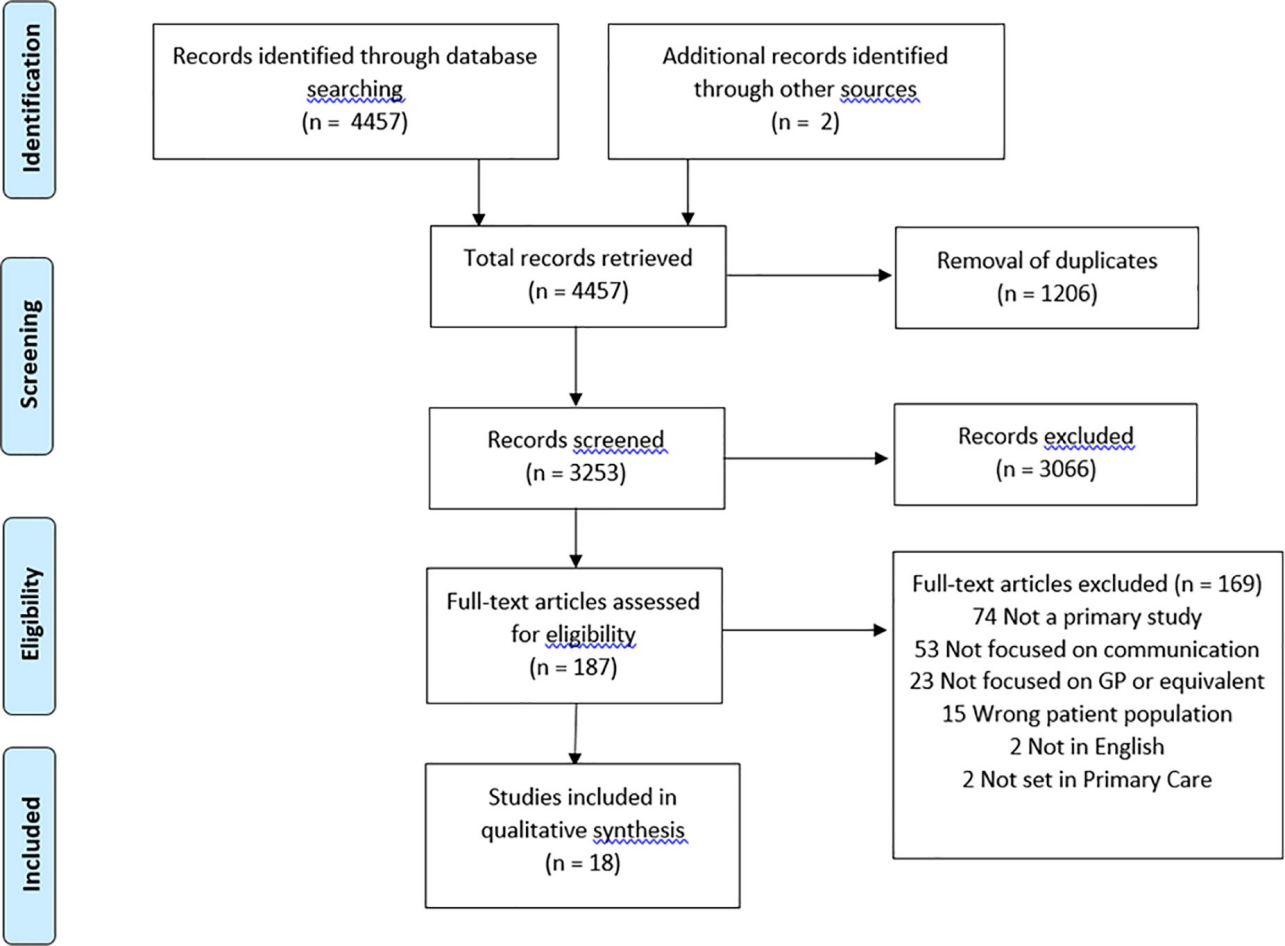
Flow diagram of systematic scoping review.

The initial search generated 4457 records, of which 18 papers met the study criteria for inclusion (Fig 1).

### Overview of included studies

Included studies are summarized in Table 2. Five studies originated from the United States of America [21–25], five from Canada [10, 26–29], six from Australia [11, 30–34], and one each from New Zealand [35] and Ireland [9]. Year of publication spanned 1999 to 2015. Seven studies were cross-sectional surveys [9, 21, 28, 32–35], six studies were pre/post evaluations of improvement or education programmes [22, 23, 25, 26, 29, 30], and five were primary qualitative studies [10, 11, 24, 27, 31]. Eleven studies focused on FtD in the specific setting of cognitive impairment [9, 11, 21, 23, 24, 26, 28–30, 33, 35], five studies on the general assessment of FtD in older people (but included data on cognitive impairment) [10, 22, 31, 32, 34], and two studies on the broader management of dementia but included data on FtD [25, 27]. Four studies provided patient or care-giver views [11, 21, 24, 30], while fifteen examined PCPs’ views.

**Table 2:** Characteristics of included studies.

Study	Aim	Study Design	Country	Participants	Sample Size
**Adler et al., 1999 [21]**	To understand the importance of driving in the lives of older adults with dementia	Cross-sectional	America	People with dementia (n = 75) and collateral sources familiar with their driving (n = 75)	n = 150
**Byszewski et al., 2003 [26]**	To examine the effect of the Driving and Dementia Toolkit on physician knowledge and confidence gained in undertaking an office assessment of driving skills	Pre/post questionnaires	Canada	Family physicians	n = 145
**Carmody et al., 2014 [30]**	To evaluate how a self-administered decision aid contributed to decision making about driving retirement by individuals living with dementia	Pre/post questionnaires	Australia	Drivers with dementia	n = 12
**Doherty et al., 2015 [9]**	To establish the general practice experience of assessing patients with cognitive impairment for driving fitness, examine the GPs attitude to this role, and investigate what factors influence GPs in this decision-making process	Cross-sectional survey	Ireland	General Practitioners	n = 125
**Friedland et al., 2006 [10]**	To examine perceptions of family physicians regarding their role of monitoring seniors’ driving and understand their perspective on both the informal and legislated aspects of their role	Qualitative focus groups	Canada	Family physicians	n = 20
**Hill et al., 2013 [22]**	To assess a curriculum that trains health professionals to increase their awareness, screening, management, and reporting of age-related driving impairments	Pre/post questionnaires	America	Healthcare professionals including General Practitioners, Occupational Therapists. Nurse Practitioners, Physician Assistants	n = 1202
**Hoggarth, 2013 [35]**	To assess how GPs in Canterbury determine the driving ability of their older patients with cognitive impairments	Cross-sectional survey	New Zealand	General Practitioners	n = 514
**Hum et al., 2014 [27]**	To explore perceived roles and attitudes towards the provision of dementia care from the perspectives of family physicians and specialists	Qualitative interviews	Canada	Family physicians (n = 6) and hospital specialists (n = 6)	n = 12
**Johnson et al., 2013 [11]**	To investigate the views of older people with mild cognitive impairment about decision making on driving cessation	Qualitative interviews	Australia	People with suspected cognitive impairment	n = 7
**Jones et al., 2012 [31]**	To explore GP perspectives regarding assessing fitness to drive in older and functionally impaired patients	Qualitative interviews and one focus group	Australia	General Practitioners	n = 13
**Lipski, 2002 [32]**	To investigate the attitudes of General Practitioners to older drivers on the New South Wales Central Coast.	Cross-sectional survey	Australia	General Practitioners	n = 173
**Meuser et al., 2006 [23]**	To develop and evaluate a multimedia workshop curriculum to educate physicians and other health professionals about (a) driving-related assessment in older adults with dementia, and (b) strategies to encourage driving retirement for impaired individuals	Pre/post questionnaires at 4 time points	America	Licensed health professionals	n = 190
**Moorhouse et al., 2011 [28]**	To assess perceived barriers to addressing driving safety in dementia among Nova Scotian primary care physicians and to determine whether these barriers differ between urban and rural physicians or according to years of practice	Cross-sectional survey	Canada	Primary Care Physicians	n = 134
**Moorhouse and Hamilton, 2014 [29]**	To assess the impact of a provincial Web-based resource (www.notifbutwhen.ca) regarding driving cessation in dementia aimed towards primary care physicians	Pre/post questionnaires	Canada	Primary Care Physicians	n = 134 /n = 113
**Perkinson et al., 2005 [24]**	To examine beliefs and responses to the issue of driving and Alzheimer’s Dementia among key stakeholder groups, including views on the circumstances that either allow persons with dementia to continue driving or prompt them to retire, beliefs regarding the identification and management of unsafe drivers with AD and the perceived barriers to and successful strategies for achieving driving cessation when appropriate	Qualitative focus groups	America	General Practitioners (n = 8); Drivers with very mild to mild cognitive impairment (n = 9); Former drivers with very mild to mild cognitive impairment (n = 5); Family caregivers of drivers (n = 9); Family caregivers of former drivers (n = 5); Advocates (n = 10); Non Physician Healthcare Staff (n = 8); Transport and law enforcement professionals (n = 8); Geriatricians and Neurologists (n = 6)	n = 68
**Reuben et al., 2010 [25]**	To determine whether a practice redesign intervention coupled with referral to local Alzheimer's Association chapters can improve the quality of dementia care	Pre/post medical intervention audits	America	Two community-based physician practices and patients aged 75+ with dementia.	N = 5
**Snellgrove & Heckler, 2002 [33]**	To investigate the attitudes, knowledge, and self-reported clinical practices of GPs in South Australia regarding driving and dementia	Cross-sectional survey	Australia	General Practitioners	n = 1,000 (approx)
**Wilson and Kirby, 2008 [34]**	To investigate individual differences in GP knowledge, procedures and opinions of older driver assessments	Cross-sectional survey	Australia	General Practitioners	n = 204

### PCPs content to discuss but not assess driving ability in setting of cognitive impairment

PCPs were generally content to discuss driving with their cognitively-impaired patients and act as a first-point of contact for patients with concerns, but they disliked the “*emotionally-charged task*” of actually assessing or determining patients’ FtD [9, 10, 27, 28, 31, 33, 34]. In North American and Australian studies, PCPs voiced a preference for an overall shift in responsibility for assessment to third parties such as physicians within Ministries of Transportation, hospital-based geriatric programs or occupational therapists [10, 27, 28, 31, 33, 34].

Less than a third of sampled Canadian PCPs were comfortable with their ability to assess FtD [26, 28, 33, 34], and almost 70% at least sometimes avoided discussions about driving [28]. Similar findings were observed in the US where PCPs self-rated their confidence in FtD assessments in cognitive impairment as 4.3/7 [23] and almost half (48%) of Californian PCPs reported an absence of confidence in their skills [22]. In New Zealand, the majority of PCPs were “not so confident” about driving in the setting of cognitive impairment [35].

PCPs’ discomfort was associated with infrequent screening of older drivers’ cognition: the majority of New Zealand PCPs only “sometimes” screened cognition for older drivers [35]; three Australian studies indicated that as few as 16–32% of PCPs routinely screened cognition during FtD assessment [32–34]; and 62% of American PCPs admitted “rarely” or “never” screening their elderly patients for functional/ cognitive impairments [22]. Similarly, data on routine care for patients with dementia showed that enquiries about driving were made by only a third (38%) of PCPs [23].

Years of experience as a physician was positively associated with having routine discussions about driving, more lengthy discussions about driving cessation [28], and physician confidence with driving assessment [26]. For example, the majority of surveyed Irish PCPs had been qualified for more than twenty years and this group reported high levels of confidence assessing fitness to drive despite feeling inadequately resourced to do so [9].

### Reasons for PCPs’ discomfort

**Fig 2:**
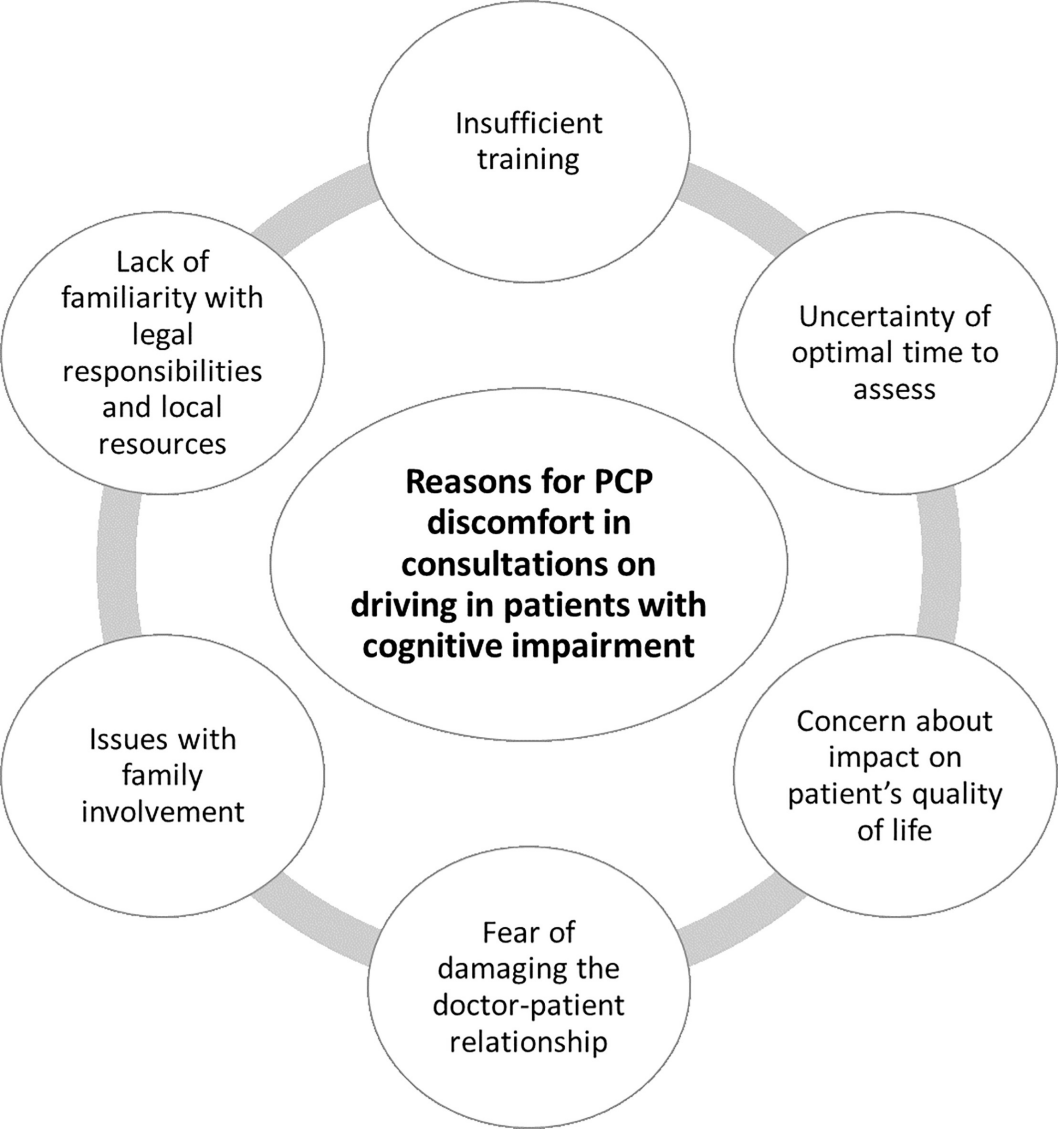
Reasons for PCP discomfort in consultations on driving in patients with cognitive impairment.

Reasons for PCPs’ discomfort assessing FtD in cognitive impairment were evident in almost all included studies and are summarised below and in Fig 2.

#### Insufficient training

Studies showed that PCPs perceived that they lacked appropriate training: over half (59%) of surveyed Australian PCPs reported insufficient training in the medical assessment of drivers and driving competency [32] while Canadian PCPs stated that they felt undermined by their lack of training in assessing patients’ ability to drive [10]. Specific areas of difficulty were: 40% of surveyed Canadian PCPs expressed difficulties with decision-making on FtD [28], a similar proportion (46%) of Australian PCPs reported difficulties distinguishing normal ageing from early dementia [33] and 60% of Irish PCPs desired additional training in assessment of cognition relevant to driving [9]. PCPs in some studies felt they were too pressed for time during consultations to undertake satisfactory reviews of driving ability [10], and called for brief desktop references to guide in-office assessment and decision-making to facilitate greater efficiency [10, 27, 31, 33, 35].

#### Lack of familiarity with legal responsibilities and local resources

Distinct from lack of training, PCPs in studies in multiple jurisdictions reported poor familiarity with legal obligations and responsibilities for notifying licensing authorities [9, 10, 22, 23, 28, 33–35] and a lack of knowledge of local resources and supports for patients [9, 23, 25]. Canadian PCPs described themselves as “*reluctant regulators*” [10], with over a third (36%) reporting lack of familiarity with standards and guidelines [28]. Uncertainties about the right or the responsibility to breach patient confidentiality were also apparent in multiple studies [22, 23, 31, 33].

#### Fear of damaging the doctor-patient relationship

Qualitative data revealed that PCPs saw raising the topic of driving in the setting of cognitive impairment as something that *“can completely destroy the therapeutic relationship with the patient”* [27] as well as potentially alienating the patient [10, 22, 24, 31]. The qualitative findings were supported across the survey data, with 43 to 48% of PCPs agreeing that the potential negative impact on doctor-patient relationship was a barrier to FtD assessment for patients with dementia [9, 23, 28, 29, 33, 34]. PCPs’ fears in this regard were not unfounded: participants in two studies reported break downs in the doctor-patient relationship that led to the patient switching physicians [9, 35].

#### Concern about impact on patient’s quality of life

PCPs’ concern about the impact of driving cessation on the patient’s quality of life, which included reduced self-esteem and dependence in daily activities, also led them to avoid introducing the topic in patients with earlier cognitive impairment, especially in areas lacking alternative community transport services [24, 31, 33, 34]. Others expressed concern that patients who ‘*fired*’ them might forego medical care of their dementia and other conditions [27]. The findings of one study suggested that by demonstrating an awareness of the negative impact associated with driving cessation, PCPs could mitigate some of the bad feeling between doctor and patient: “*Addressing the negative issues shows you are aware of impact*” [34].

#### Issues with family involvement

PCPs reported that the impact of FtD in cognitive impairment as “*a big*, *ugly problem”* could extend beyond the doctor-patient relationship to the PCP’s relationship with entire families: *“families and patients get mad when driving is taken away”* [27]. A perceived lack of support from the family or caregivers was viewed by participants in many studies as an additional barrier to speaking to a person with dementia about driving [10, 28, 29, 31]. However, PCPs also saw that concerns expressed by family members was a useful trigger for further evaluation [10], and gaining family support could facilitate this process [24, 31].

#### Uncertainty of optimal time to assess

PCPs tended to conduct initial driving screening soon after a diagnosis of cognitive impairment or dementia had been made [28] with the transition from very mild cognitive impairment to mild cognitive impairment identified as a key time to raise the topic of driving [23] and as an opportunity to “plant the seed” for planning of driving retirement [24]. However, introducing the topic too early led to its own problems: in one Canadian study, PCPs found assessment of the impact of early cognitive impairment on driving difficult because “*… seniors are well socialized to a medical visit and can cover up their deficits very nicely*”[10] while Australian PCPs felt patients with early dementia had “..*the least insight into their driving inability*” [31]. Further, it was felt that patients’ may not take advantage of early support due to the perception that they did not need it then [25].

### Patient and carer perspective

The key finding from three studies with older people with cognitive impairment was ‘*maintaining agency’*: people ideally wanted to decide when they should stop driving themselves but they were theoretically prepared to accept their PCP’s advice and family input [11, 21, 24]. Patient participants in an Australian qualitative study accepted that they would have to stop driving at some stage, and anticipated that their PCP would advise them when they were no longer fit to drive, but the majority of patients and caregivers in a Canadian survey believed, mistakenly, that patients could continue driving through the course of their illness [21]. Participants in the three studies saw referral for assessment as acceptable or even desirable to settle any dilemmas or uncertainties about FtD [11, 21, 24]. Male respondents linked the loss of capacity to drive with a loss of male identity, leading to a suggestion to PCPs to acknowledge this issue explicitly when dealing with male patients [11].

### Value of interventions

An overview of interventions used in the six intervention studies is provided in Table 3, with material which may support PCPs’ approach to the topic of driving in consultations with patients with cognitive impairment highlighted in the final column. Three of the four interventions aimed at increasing PCP confidence in; knowledge of; and screening for driving ability in cognitive impairment were found to be successful [22, 23, 26]. These three interventions shared some common features: they provided an overview of information on local and regional resources, legal requirements, and assessment strategies to support PCPs’ approach; they focused on what physicians can accomplish in an office visit alone; and information was provided succinctly through short in-person lectures or workshops [22, 23] or via a posted booklet [26]. The fourth intervention, a web-based education campaign, was less successful [29]. Engagement with the on-line material was low, and while participants reported being less likely to avoid discussions about driving with patients, there was no significant change in self-rated comfort assessing FtD in dementia, and the proportion who felt ill-equipped remained high (83%). A fifth intervention focused on practice redesign to improve dementia care and was associated with increased referral to local Alzheimer’s Association chapters, which in turn increased the likelihood of referred patients receiving counselling on driving cessation and improved the quality of counselling about driving that they received [25].

**Table 3:** Description of interventions.

**Study**	**Intervention**	**Key findings**	**Generic tips for PCPs specific to communication**
**Byszewski et al., 2003 [26]**	The Driving and Dementia Toolkit • designed to respond to the need for information to assist family physicians in the office assessment of driving skills, in communicating the results of the assessment to patients and their caregivers, and in linking patients and family members with the appropriate community resources • consists of background information, algorithm of local resources, forms to access these services, screening questions about older drivers’ safety, patient-related information, and frequently asked questions • material was printed as a booklet, and posted to family physicians	The toolkit significantly improved PCPs self-reported knowledge and confidence for assessing driving capacity in people with dementia in primary care by: • increasing awareness of specialist and government approved services available (89.7%) • increasing familiarity with appropriate questions to ask patients (68–98%) and their caregivers (60–97%) when assessing driving ability	Questions to ask patients • do you think you are a safe driver • do you restrict driving to familiar areas/routes • do other drivers honk at you or show irritation • have you noticed any change in your driving skills Questions to ask caregivers: • does the patient avoid driving at night • has the patient received any traffic violations • does the person need a co-pilot to alert them of potentially hazardous events or conditions • do you feel uncomfortable being a passenger when the patient is driving
**Carmody et al., 2014 [30]**	The Driving With Dementia Decision Aid (DDDA) to guide patients through: • clarification of their decision and values • decisional needs and support • consideration of the options • advising others of one’s decision. Link to Dementia and Driving: a decision aid	The DDDA improved patients’ knowledge and satisfaction with decisions regarding driving retirement by • Increasing knowledge from 5.3 to 5.8 (out of 10) • Changing their decisions regarding driving • Reducing decisional conflict from 22.5 to 7.5 (out of 100) Patients felt that the DDDA would be a good tool to start conversations with others about their driving.	Move focus away from assessment of FtD, to focus instead on facilitating planning for driving retirement with patients recently diagnosed with dementia. Aim to engage and assist people recently diagnosed with dementia in their decisions and plans for driving retirement, thereby protecting patient agency while also maintaining public road safety.
**Hill et al., 2013 [22]**	One hour seminar on age-related driving impairments including: • Statistics on older drivers, vision, frailty and cognitive decline • Implementation and interpretation of approved screening tools • Pocket guide with algorithm for outcomes of screening, counselling patients and reporting to driving authorities • Resources and when to refer to occupational therapists, driving rehab specialists etc.	The training programme increased: • confidence in screening older people for age related driving impairments (to 72%) • intent to screen (to 55%) • understanding of the law (92%) • understanding of medical conditions and medications that might impair ability to drive (92%) Mandatory reporting was perceived to: (1) protect safety of patients (91%); (2) increase willingness to discuss driving with patients (59%); (3) protect PCPs from liability; (4) have the potential to alienate patients.	Promote general health and ensure optimal medication use to best support on-going driving (i.e. vision, range of motion, use lowest effective dose of medications etc.) Be familiar with local resources and regional legal requirements
**Meuser et al., 2006 [23]**	Two hour multimedia workshop covering • the approach to evaluating the driver with dementia • counselling the patient and family • state reporting procedures for impaired drivers • web-based resources • local and national referral sources Link to resource booklet “At the Crossroads: Family Conversations about Dementia and Driving”	The workshop was associated with • improvements in PCPs’ self-rated confidence from 4.3/7 to 6.9/7 with sustained improvement at three and twelve months • reduced confusion about reporting procedures, uncertainty about protection against confidentiality breaches, and fear of damaging the doctor-patient relationship	Where impairment is very mild, advise the person and family that driving cessation will be required eventually. Follow up every 6–12 months. Where mild, educate the patient and family that the advancing impairment will likely necessitate retirement from driving in 6–18 months. Recommend common sense restrictions to reduce risk e.g. avoiding bad weather, night-time, rush hour driving and recommend that they begin to develop an alternate transport plan. Moderate to Severe: Recommend immediate retirement from driving. Work with patient and family to develop and implement a plan for driving cessation and alternate transportation. Enlist help of others to ensure active acceptance of the plan.
**Moorhouse and Hamilton, 2014 [29]**	Launch of a web-based campaign and resource (www.notifbutwhen.ca) to guide physicians through the process of driving cessation from the time that cognitive concerns are first noticed through to when dementia precludes safe driving, including: • summary of evidence on driving safety in dementia • in-office driving assessments and national guidelines regarding driving safety in dementia • referral forms for local driving assessment agencies • algorithms for determining when on-road assessment may be needed • step-by-step guides to the process once concerns are raised to the provincial Registry of Motor Vehicles • printable information sheets and checklists for caregivers	After the web-resource was launched participants were • more likely to address FtD as part of routine dementia care • less likely to wait for concerns to be resented by family members before initiative discussions about driving • less likely to report avoiding discussions about driving (69% to 53%) • less likely to cite family resistance or a lack of resources to offer patient/families as barriers There was no significant change in physician’s comfort assessing fitness to drive (40% to 36%).	Increasing familiarity with local resources for driving assessment and supports for patients and caregivers can facilitate discussions about driving.
**Reuben et al., 2010 [25]**	ACOVE-2 intervention: • a practice redesign intervention (involving screening, efficient collection of clinical data, medical record prompts, patient education/ empowerment materials, and physician decision support/education) coupled with referral to local Alzheimer's Association chapters	This intervention led to more patients with dementia being referred to local Alzheimer's Association chapters. Referred patients had higher quality scores (65% versus 41%) and better counselling about planning for driving cessation (50% versus 14%).	Consider referral of all patients with dementia to local Alzheimer Associations for provision of support and information regarding driving cessation.

The sixth intervention aimed to support people with dementia to engage in decision-making on driving retirement [30]. In this pilot study, drivers with dementia (n = 12) reported improved knowledge, higher satisfaction with decisions regarding driving retirement, and less decisional conflict after reading a clearly worded decision-aid.

## Discussion

This scoping review was undertaken to describe, synthesize, and interpret literature on consultations between PCPs and patients with cognitive impairment and their caregivers about FtD. The synthesized data highlight why PCPs encounter challenges and sometimes avoid these consultations. Whereas PCPs view this doctor-patient interaction as potentially contentious, the patient literature suggests the potential benefits of re-framing FtD consultations as a proactive and collaborative discussion between PCPs and persons with cognitive impairment. Data also support the need for professional educational modules that are succinct, closely aligned with the challenges of practice and include easily retrievable information on local resources for driving assessment, patient support and legal responsibilities. Addressing these knowledge gaps will help to build PCPs’ confidence in approaching the topic of driving in consultations.

### Implications for practice

Stopping driving can limit an older person’s independence and is an independent risk factor for entry to a nursing home [3], but these negative consequences must be weighed up against the higher accident rates experienced by older drivers with cognitive impairment and the risks to other road users [36–38]. Our findings, particularly those in Table 3, outline specific communication techniques that PCPs can use to introduce the topic of driving in consultations with people with cognitive impairment (and/or their caregivers). Rather than seeing these discussions as a threat, these techniques may help to harness the strengths of the longitudinal doctor-patient relationship to better deliver patient-focused driving advice. Promotion of early and open conversation about FtD by healthcare professionals, patient advocacy groups and the lay media may prompt and encourage better discussions about driving between patients and PCPs [10, 29]. These communication techniques may also have broader application for PCPs who are trying to introduce the topic of cognitive impairment with patients who appear to lack awareness of cognitive deficits they are manifesting.

### Strengths and limitations

Key strengths are the systematic search, inclusion of mixed study designs and the multidisciplinary team. Our main reason for excluding papers was the lack of empirical evidence: we found much has been written on how PCPs should conduct FtD consultations, but empirical evidence on this matter is lacking. While no included study was “fatally flawed”, study quality was generally low, attesting that this is an area worthy of much more research endeavour. Specifically, we identified in the available literature a dearth of evidence on the lived experience of patients and caregivers who had encountered FtD consultations in primary care (whether negative or positive); a lack of experimental evidence for the effect of PCP education or training interventions on patient experience; and little consideration for how third-party assessment can be integrated into patient care without impacting on continuity and patient-centredness.

## Conclusion

The increasing number of older people affected by cognitive impairment, for whom driving may be a concern, has implications for primary care practice. Addressing the reasons for PCPs’ lack of comfort in dealing with this issue is essential in order for them to better engage with proactive, collaborative discussion with patients on plans and preferences for driving cessation.

## Supporting information

S1 PRISMA checklist (DOC)

S1 Table Medline search strategy. (DOCX)

S2 Table Search dates for each database. (DOCX)

## References

[1] Dickerson AE, Molnar LJ, Eby DW, Adler G, Bedard M, Berg-Weger M, et al Transportation and aging: a research agenda for advancing safe mobility. The Gerontologist. 2007;47(5):578–90. 17989400 10.1093/geront/47.5.578

[2] Liddle J, Carlson G, McKenna K. Using a matrix in life transition research. Qualitative health research. 2004;14(10):1396–417. 10.1177/1049732304268793 15538007

[3] Breen DA, Breen DP, Moore JW, Breen PA, O'Neill D. Driving and dementia. BMJ. 2007;334(7608):1365–9. 10.1136/bmj.39233.585208.55 17600026 PMC1906602

[4] Prince MJ, Wimo A., Guerchet M. M., Ali G. C., Wu Y-T., & Prina M. World Alzheimer Report 2015. The Global Impact of Dementia. An analysis of prevalence, incidence, cost and trends Alzheimer’s Disease International (ADI), London; 2015.

[5] Bogner HR, Straton JB, Gallo JJ, Rebok GW, Keyl PM. The role of physicians in assessing older drivers: barriers, opportunities, and strategies. The Journal of the American Board of Family Practice. 2004;17(1):38–43. 15014051 10.3122/jabfm.17.1.38 PMC2804856

[6] Jang RW, Man-Son-Hing M, Molnar FJ, Hogan DB, Marshall SC, Auger J, et al Family physicians' attitudes and practices regarding assessments of medical fitness to drive in older persons. J Gen Intern Med. 2007;22(4):531–43. 10.1007/s11606-006-0043-x 17372806 PMC1829420

[7] Moore V, Cahill S. Diagnosis and disclosure of dementia—a comparative qualitative study of Irish and Swedish General Practitioners. Aging Ment Health. 2013;17(1):77–84. 10.1080/13607863.2012.692763 22690732

[8] Berkhof M, van Rijssen HJ, Schellart AJ, Anema JR, van der Beek AJ. Effective training strategies for teaching communication skills to physicians: an overview of systematic reviews. Patient education and counseling. 2011;84(2):152–62. 10.1016/j.pec.2010.06.010 20673620

[9] Doherty U, Hawke AL, Kearns J, Kelly M. Fitness to drive in cognitive impairment—a quantitative study of GPs' experience. Irish medical journal. 2015;108(4):112–4. 26016301

[10] Friedland J, Rudman DL, Chipman M, Steen A. Reluctant Regulators: Perspectives of Family Physicians on Monitoring Seniors' Driving. Topics in Geriatric Rehabilitation. 2006;22(1):53–60.

[11] Johnson DA, Frank O, Pond D, Stocks N. Older people with mild cognitive impairment—their views about assessing driving safety. Australian family physician. 2013;42(5):317–20. 23781534

[12] Arksey H, O'Malley L. Scoping studies: towards a methodological framework. International Journal of Social Research Methodology. 2005;8(1):19–32.

[13] Murphy E, Froggatt K, Connolly S, O'Shea E, Sampson EL, Casey D, et al Palliative care interventions in advanced dementia. The Cochrane database of systematic reviews. 2016;12:Cd011513 10.1002/14651858.CD011513.pub2 27911489 PMC6463843

[14] Chandler MJ, Parks AC, Marsiske M, Rotblatt LJ, Smith GE. Everyday Impact of Cognitive Interventions in Mild Cognitive Impairment: a Systematic Review and Meta-Analysis. Neuropsychology review. 2016;26(3):225–51. 10.1007/s11065-016-9330-4 27632385 PMC5048589

[15] Hoekstra RA, Heins MJ, Korevaar JC. Health care needs of cancer survivors in general practice: a systematic review. BMC Family Practice. 2014;15:94–. 10.1186/1471-2296-15-94 24885266 PMC4031325

[16] Derksen F, Bensing J, Lagro-Janssen A. Effectiveness of empathy in general practice: a systematic review. The British journal of general practice: the journal of the Royal College of General Practitioners. 2013;63(606):e76–84. 23336477 10.3399/bjgp13X660814 PMC3529296

[17] Baker A, Unsworth CA, Lannin NA. Determining fitness to drive: A systematic review of the methods and assessments used after mild traumatic brain injury. British Journal of Occupational Therapy. 2015;78(2):73–84.

[18] George S, Crotty M, Gelinas I, Devos H. Rehabilitation for improving automobile driving after stroke. The Cochrane database of systematic reviews. 2014(2):Cd008357 10.1002/14651858.CD008357.pub2 24567028 PMC6464773

[19] Pluye P, Hong QN. Combining the Power of Stories and the Power of Numbers: Mixed Methods Research and Mixed Studies Reviews. Annual Review of Public Health. 2014;35(1):29–45. 10.1146/annurev-publhealth-032013-182440 24188053

[20] Dixon-Woods M, Cavers D, Agarwal S, Annandale E, Arthur A, Harvey J, et al Conducting a critical interpretive synthesis of the literature on access to healthcare by vulnerable groups. BMC medical research methodology. 2006;6:35 10.1186/1471-2288-6-35 16872487 PMC1559637

[21] Adler G, Rottunda S, Kuskowski M. Dementia and Driving. Clinical Gerontologist. 1999;20(2):23–34.

[22] Hill LL, Rybar J, Styer T. Evaluation of curriculum to improve health professionals' ability to manage age-related driving impairments. Accident; analysis and prevention. 2013;61:222–32. 10.1016/j.aap.2012.09.026 23127605

[23] Meuser TM, Carr DB, Berg-Weger M, Niewoehner P, Morris JC. Driving and dementia in older adults: Implementation and evaluation of a continuing education project. The Gerontologist. 2006;46(5):680–7. 17050760 10.1093/geront/46.5.680

[24] Perkinson MA, Berg-Weger ML, Carr DB, Meuser TM, Palmer JL, Buckles VD, et al Driving and dementia of the Alzheimer type: beliefs and cessation strategies among stakeholders. The Gerontologist. 2005;45(5):676–85. 16199403 10.1093/geront/45.5.676

[25] Reuben DB, Roth CP, Frank JC, Hirsch SH, Katz D, McCreath H, et al Assessing care of vulnerable elders—Alzheimer's disease: a pilot study of a practice redesign intervention to improve the quality of dementia care. Journal of the American Geriatrics Society. 2010;58(2):324–9. 10.1111/j.1532-5415.2009.02678.x 20374405 PMC3667696

[26] Byszewski AM, Graham ID, Amos S, Man-Son-Hing M, Dalziel WB, Marshall S, et al A continuing medical education initiative for canadian primary care physicians: the driving and dementia toolkit: a pre- and postevaluation of knowledge, confidence gained, and satisfaction. Journal of the American Geriatrics Society. 2003;51(10):1484–9. 14511173 10.1046/j.1532-5415.2003.51483.x

[27] Hum S, Cohen C, Persaud M, Lee J, Drummond N, Dalziel W, et al Role expectations in dementia care among family physicians and specialists. Canadian geriatrics journal: CGJ. 2014;17(3):95–102. 10.5770/cgj.17.110 25232368 PMC4164682

[28] Moorhouse P, Hamilton L, Fisher T, Rockwood K. Barriers to assessing fitness to drive in dementia in nova scotia: informing strategies for knowledge translation. Canadian geriatrics journal: CGJ. 2011;14(3):61–5. 10.5770/cgj.v14i3.7 23251315 PMC3516349

[29] Moorhouse P, Hamilton LM. Not if, but when: impact of a driving and dementia awareness and education campaign for primary care physicians. Canadian geriatrics journal: CGJ. 2014;17(2):70–5. 10.5770/cgj.17.109 24883165 PMC4038538

[30] Carmody J, Potter J, Lewis K, Bhargava S, Traynor V, Iverson D. Development and pilot testing of a decision aid for drivers with dementia. BMC medical informatics and decision making. 2014;14:19 10.1186/1472-6947-14-19 24642051 PMC3999924

[31] Jones K, Rouse-Watson S, Beveridge A, Sims J, Schattner P. Fitness to drive—GP perspectives of assessing older and functionally impaired patients. Australian family physician. 2012;41(4):235–9. 22472687

[32] Lipski PS. A survey of general practitioners' attitudes to older drivers on the New South Wales Central Coast. Australasian Journal on Ageing. 2002;21(2):98–100.

[33] Snellgrove CA, Hecker JR. Driving and dementia: General practitioner attitudes, knowledge and self-reported clinical practices in South Australia. Australasian Journal on Ageing. 2002;21(4):210–2.

[34] Wilson LR, Kirby NH. Individual differences in South Australian general practitioners' knowledge, procedures and opinions of the assessment of older drivers. Australas J Ageing. 2008;27(3):121–5. 10.1111/j.1741-6612.2008.00304.x 18713171

[35] Hoggarth PA. Diagnosis of cognitive impairment and the assessment of driving safety: a survey of Canterbury GPs. The New Zealand medical journal. 2013;126(1387):87–97. 24362737

[36] Morgan R, King D. The older driver—a review. Postgraduate Medical Journal. 1995;71(839):525–8. 7479463 10.1136/pgmj.71.839.525 PMC2398236

[37] Dobbs BM, Carr DB, Morris JC. Evaluation and management of the driver with dementia. The neurologist. 2002;8(2):61–70. 12803692 10.1097/00127893-200203000-00001

[38] Martin AJ, Marottoli R, O'Neill D. Driving assessment for maintaining mobility and safety in drivers with dementia. The Cochrane database of systematic reviews. 2009(1):Cd006222 10.1002/14651858.CD006222.pub2 19160270

