# Supplementary material for: Consultations on driving in people with cognitive impairment in primary care: A scoping review of the evidence
Source: PLoS One. 2018 Oct 15;13(10):e0205580. doi: 10.1371/journal.pone.0205580 (PMC6188864; doi:10.1371/journal.pone.0205580)
Supplement: S1 Table — (DOCX) [file pone.0205580.s002.docx]

**Supplementary information. S1 Table: Medline Search Strategy**

| S14 | S9 AND S10 AND S11 AND S12 |
| --- | --- |
| S13 | S9 AND S10 AND S11 AND S12 |
| S12 | S7 OR S8 |
| S11 | S5 OR S6 |
| S10 | S3 OR S4 |
| S9 | S1 OR S2 |
| S8 | (MH "Communication") OR (MH "Communication Barriers") OR (MH "Nonverbal Communication") OR (MH "Health Communication") OR (MH "Persuasive Communication") OR (MH "Communication Methods, Total") OR (MH "Communication Aids for Disabled") OR (MH "Interpersonal Relations") OR (MH "Risk Assessment") OR (MH "Needs Assessment") OR (MH "Symptom Assessment") OR (MH "Geriatric Assessment") OR (MH “Policy”) OR (MH "Guideline") OR (MH "Practice Guideline") OR (MH "Guideline Adherence") OR (MH "Guidelines as Topic") OR (MH "Standard of Care")(MH "Communication") OR (MH "Communication Barriers") OR (MH "Nonverbal Communication") OR (MH "Health Communication") OR (MH "Persuasive Communication") OR (MH "Communication Methods, Total") OR (MH "Communication Aids for Disabled") OR (MH "Interpersonal Relations") OR (MH "Risk Assessment") OR (MH "Needs Assessment") OR (MH "Symptom Assessment") OR (MH "Geriatric Assessment") OR (MH “Policy”) OR (MH "Guideline") OR (MH "Practice Guideline") OR (MH "Guideline Adherence") OR (MH "Guidelines as Topic”) |
| S7 | communicat* OR discuss* OR talk* OR conversation* OR assess* OR report* OR dialog* OR interfac* OR meet* OR tell* OR liais* OR convey* OR impart* OR relay* OR transfer* OR divulg* OR disclos* OR mention* OR announc* OR recount* OR transmit* OR “make known” OR “made known” OR “pass* on” OR “hand* on” OR report* OR relat* OR present* OR connect* OR inform* OR interact* OR reveal* OR suggest* OR transfer* OR hint* OR advis* OR advice OR imply* OR declar* OR discover* OR enlighten* OR rais* OR strateg* OR approach* OR guid* OR policy* OR procedure* OR protocol* OR consult* OR decision* OR technique* OR pretend* OR “cover-up” OR fabricat* OR lie OR falsif* OR deny OR denial OR truth OR hid*communicat* OR discuss* OR talk* OR conversation* OR assess* OR report* OR dialog* OR interfac* OR meet* OR tell* OR liais* OR convey* OR impart* OR relay* OR transfer* OR divulg* OR disclos* OR mention* OR announc* OR recount* OR transmit* OR “make known” OR “made known” OR “pass* on” OR “hand* on” OR report* OR relat* OR present* OR connect* OR inform* OR interact* OR reveal* OR suggest* OR transfer* OR hint* OR advis* OR advice OR imply* OR declar* OR discover* OR enlighten* OR rais* |
| S6 | (MH “Automobiles”) OR (MH “Automobile Driving”) OR (MH “Automobile Driver Examination”) OR (MH “Accidents, Traffic”) OR (MH Motor Vehicles) |
| S5 | driv* OR car OR cars OR automobile* OR traffic OR transport* OR vehicle* OR motor* OR (road N3 assessment*) |
| S4 | (MH “General Practitioners”) OR (MH “General Practice”) OR (MH Primary Health Care) OR (MH “Physicians, Primary Care”) OR (MH “Ambulatory Care”) OR (MH “Family Practice”) OR (MH “Physicians, Family”) |
| S3 | “general practi*” OR GP* OR “primary care” OR “family medicine” OR doctor* OR physician* OR “primary healthcare” OR “primary health-care” OR “primary health care” OR “family practice” OR “family health” OR “community care” OR “ambulatory care” |
| S2 | (MH "Dementia") OR (MH "Frontotemporal Dementia") OR (MH "Dementia, Vascular") OR (MH "Dementia, Multi-Infarct") OR (MH "AIDS Dementia Complex") OR (MH "Alzheimer Disease") OR (MH "Lewy Body Disease") OR (MH “Mild Cognitive Impairment”) OR (MH “Cognition Disorders”) OR (MH “Cognitive Aging”) |
| S1 | dement* OR alzheimer* OR “cognitive impairment*” OR “cognitive disorder*” OR *senil* OR “lew* N2 bod*” OR FTLD OR FTD OR frontotemporal OR deliri* OR "Wernicke Encephalopathy" OR amnestic OR amnesia OR "Cognitive Disorders" OR "chronic N2 cerebrovascular" OR "organic brain disease" OR "organic brain syndrome" OR "normal pressure hydrocephalus" OR "shunt*" OR "benign senescent forgetfulness" OR (cerebr* N2 deteriorat*) OR (cerebral* N2 insufficient*) OR "normal pressure hydrocephalus" OR "shunt*" OR "benign senescent forgetfulness" OR (cerebr* N2 deteriorat*) OR (cerebral* N2 insufficient*) OR (pick* N2 disease) OR creutzfeldt OR jcd OR cjd OR huntington* OR binswanger* OR korsako* OR delirium OR mci OR amci OR mci-a OR predement* OR pre-dement* OR pre-AD OR (cogniti* N2 (decline OR impair* OR deficit* OR deteriorate* OR disord*)) OR ((prelude OR preclinical OR pre-clinical OR prodromal OR precursor) N2 (dement* OR Alzheimer* OR AD)) OR "progressive isolated aphasia" OR "grey matter" OR circumscribed brain atrophy" |
