# Supplementary material for: Consultations on driving in people with cognitive impairment in primary care: A scoping review of the evidence
Source: PLoS One. 2018 Oct 15;13(10):e0205580. doi: 10.1371/journal.pone.0205580 (PMC6188864; doi:10.1371/journal.pone.0205580)
Supplement: S2 Table — (DOCX) [file pone.0205580.s003.docx]

**Supplementary information. S2 Table: Specific search dates for each database**

| Database name | Search dates |
| --- | --- |
| Medline | 1946 - 1^st^ Dec 2016 |
| Cumulative Index to Nursing and Allied Health Literature with Full Text (via EBSCO) | 1981 - 1^st^ Dec 2016 |
| PsycINFO | 1887 - 1^st^ Dec 2016 |
| Academic Search Complete | 1997 - 1^st^ Dec 2016 |
| Psychological and Behavioural Sciences Collection | 1965 - 1st Dec 2016 |
| SocIndex | 1895 - 1^st^ Dec 2016 |
| Social Sciences FT | 1972 - 1^st^ Dec 2016 |
